# Prevalence and predictors of self-medication with antibiotics in selected urban and rural districts of the Dodoma region, Central Tanzania: a cross-sectional study

**DOI:** 10.1186/s13756-022-01124-9

**Published:** 2022-06-16

**Authors:** Richard James Mabilika, Emmanuel Mpolya, Gabriel Shirima

**Affiliations:** 1 Department of Global Health and Biomedical Sciences, Nelson Mandela African Institution of Science and Technology, P.O.Box 447, Arusha, Tanzania; 2 School of Medicine and Dentistry, University of Dodoma, P.O.Box 259, Dodoma, Tanzania

**Keywords:** Antibiotics, Self-medication, Sociodemographics, Dodoma, Drug outlet

## Abstract

**Background:**

Antibiotic resistance is a global health threat driven partly by self-medication with antibiotics (SMA). This study aims to assess the prevalence and predictors of SMA in selected rural and urban communities of the Dodoma region, Central Tanzania.

**Methods:**

This cross-sectional study was conducted in Chemba District Council (rural) and Dodoma City Council (urban) from August to November 2019 using multistage stratified random sampling. Data were collected through face-to-face interviews using structured questionnaires.

**Results:**

A total of 430 respondents were interviewed in Chemba District Council (rural) (161/430) and Dodoma City Council (urban) (269/430). The prevalence of SMA was 23.6% (38/161) among rural respondents and 23.4% (63/269) among urban respondents. The median amount of SMA in both settings was 2, while the maximum amounts were 4 and 5, respectively. SMA among rural and urban participants was associated mostly with perceived cough (76.3%/82%), body pain (71.1%/41.5%) and fever (63.2%/39.7%), and amoxicillin was the most commonly used antibiotic in both settings (47.3%/41%). Rural participants who reported a shorter perceived distance to a health care facility than to a drug outlet were 58.9% less likely to practise SMA (adjusted OR: 0.421; 95% CI: 0.388, 0.458; *p* < 0.001), whereas SMA decreased by 16.3% among urban participants who reported a shorter perceived distance to a health care facility than to a drug outlet (adjusted OR: 0.837; 95% CI: 0.755, 0.929; *p* < 0.001). SMA was 17.3% lower among farmers than among nonfarmers in the urban area (adjusted OR: 0.827; 95% CI: 0.716, 0.955; *p* = 0.01), while farming had no effect in the rural area.

**Conclusions:**

The prevalence of SMA is similar among participants in rural and urban districts. In both localities, a shorter perceived distance to a drug outlet is an independent risk factor for SMA, while having health insurance reduces the risk. Equally weighted interventions to reduce SMA are required in rural and urban communities.

**Supplementary Information:**

The online version contains supplementary material available at 10.1186/s13756-022-01124-9.

## Introduction

The World Health Organization (WHO) defines self-medication as the “use of drugs to treat self-diagnosed disorders or symptoms, or the intermittent use of prescribed drugs for chronic recurrent diseases or symptoms without consulting a prescriber” [1]. Such practices are widely reported in both high- and low-income countries [2]. Self-medication with antibiotics (SMA) is reported to be an important driver of the misuse and overuse of antibiotics and contributes greatly to adverse drug reactions, drug interactions, prolonged morbidities, and the emergence and subsequent spread of antibiotic resistance [3]. Unguided beliefs about the efficacy of antibiotics for every kind of illness have largely accelerated antibiotic-seeking behaviours; other drivers include irrational dispensing practices in community drug outlets, poor enforcement of laws on the sale and use of antibiotics and unregulated prescribing in health care facilities [4].

The consumption of antibiotics for human health is higher in low-income countries than in high-income countries, where antibiotics are used mainly in agriculture [5, 6]. In low-income countries, antibiotics for self-medication can easily be purchased from poorly regulated and widely distributed community drugstores [7]. Poor public health systems and policies, the unregulated sale of antibiotics, high prevalence of infectious diseases, limited access to appropriate antibiotics, and poor diagnostic tools are among the factors behind excessive antibiotic use and antibiotic resistance in low-income countries [8].

Self-medication with antibiotics bought from community drugstores without a valid prescription is a common practice in low-income countries [9]. In these circumstances, antibiotics are dispensed with incorrect dosages, durations and routes of administration for conditions that do not require them [10]. A review of the determinants of community antibiotic use among outpatients identified age, level of education and employment status as factors that influence antibiotic consumption [11]. In Tanzania, studies have reported household distance from a community drug outlet and a health care facility, cost of services at health care facilities, average household income and emergency illnesses as factors influencing SMA [3, 12].

Rural and urban settings have distinct socioeconomic and demographic characteristics and may show markedly different predictors of SMA. The identification of such predictors is important in tailoring interventions for the two settings. In this study, we report the prevalence and predictors of SMA in rural and urban settings in the Dodoma region of Central Tanzania.

## Methods

### Study design and settings

This community-based analytical cross-sectional study was conducted in a rural and an urban district in the Dodoma region, Central Tanzania, from August to November 2019. In this context, a rural district has a population density of fewer than 45 people per square kilometre; a lack of modern infrastructure, such as roads and railways; and a low density of shops, reflective of low household income**.** An urban district has a population density of 45 or more people per square kilometre together; modern infrastructure, such as roads and railways; and a high density of shops, reflective of high household income.

The Dodoma region is divided into 7 districts [13], which in this study were divided into rural (Chemba, Bahi, Mpwapwa, and Chamwino Districts) and urban (Dodoma City Council, Kondoa and Kongwa) categories. One representative district from each category was then randomly selected. Dodoma City Council and Chemba District Council represented the urban and rural settings, respectively. Dodoma City Council (urban) is located between 06°10′32″S and 35°44′19″E and has a population of approximately 460,000, and Chemba District Council (rural) is located between 05°14′34″S and 35°53′24″E ﻿and has a population of approximately 250,000 [14]. Dodoma City Council covers approximately 2769 square kilometres and has 4 hospitals, 13 health centres, 48 dispensaries, 364 accredited drug dispensing outlets (ADDOs), and 62 pharmacist-operated pharmacies [13]. Chemba District Council covers a total of 7653 square kilometres and has 4 health centres, 35 dispensaries and 87 ADDOs [13].

The average household size is 4.6 persons in Chemba District Council (rural) and 4.2 persons in Dodoma City Council (urban); over 70% of those residing in Chemba District Council are employed in agriculture, while only 57% of those in Dodoma City Council practise agriculture [13]. Moreover, 93% of the rural residents live in privately owned houses compared to only 50% of those in the urban district, and 71% of the urban residents have access to piped water compared to only 15% of those in the rural district [13]. The distribution of electricity is such that 32% of the households in Dodoma City Council are electrified compared to only 3.8% of the households in Chemba District Council [13].

### Study population, inclusion and exclusion criteria

The sampling frame of this study was households in both the rural and urban districts. All adults who were present during the interview and who reported that they were permanent occupants of the household were deemed fit to participate.

### Sample size calculation

The sample size calculation was based on the unknown prevalence (P) of 50% with a 95% confidence level and was calculated using the survey formula described by Kothari [15]. The degree of precision was 5%, with a design effect of 1. The response rate was 90%. There was no population correction factor since the total population in the sampling frame was well above 5000. The final sample size was 427.

### Sampling technique

The sampling technique employed was multistage stratified random sampling involving a rural and an urban district as representative strata. Rural (Chemba, Bahi, Mpwapwa, and Chamwino) and urban (Dodoma City Council, Kondoa and Kongwa) district names were each written on a piece of paper and placed in the corresponding rural or urban basket. Chemba District Council was randomly drawn from the rural strata and Dodoma City Council from the urban strata. In the second stage, probability proportionate to size sampling was used to decide the total number of households to be included in the study between the two districts. In 2016, there were 93,339 households in Dodoma City Council and approximately 47,100 households in Chemba District Council [13]. Proportionately, we needed twice as many households in Dodoma City Council as in Chemba District Council; thus, we visited 269 households in Dodoma City Council and 161 in Chemba District Council.

Households were selected according to the ICF International DHS Toolkit [16]. The full list of all occupied households was obtained from the Council offices, and households were selected using systematic selection: a random starting point was selected, and a die with the first four faces representing the four cardinal directions (1 = east, 2 = west, 3 = north and 4 = south) was tossed to determine the direction. In the selected direction, 10 households were selected, after which the die was tossed again to determine the next direction. From each household, an adult aged 18 years or more was randomly selected for the interview. When more than one able-bodied adult was present in the household, priority was given to the head of the household or any other person acting in a similar capacity at the moment, regardless of sex.

### Data collection

Data were collected using an Open Data Kit (ODK) digital questionnaire (Additional file 1). The questionnaire was adopted from a similar study [17] and modified to incorporate the antibiotics commonly used for self-medication and the perceived disease conditions that prompt SMA as well as rural and urban variables. The modified English questionnaire was translated into Swahili (Additional file 2) for easy comprehension among respondents and data collectors, and its suitability was tested in a pilot study before it was adopted for use in this study.

The questionnaire had both closed-ended and open-ended questions that inquired about the sociodemographic information of the respondents (age, sex, education, district of residence and occupation), their history of SMA in the previous year, conditions prompting SMA, sources of antibiotics, awareness of antibiotic resistance, perceived household distance from health care facilities (hospital/health centre/dispensary) and community drug outlets and types of antibiotics used. For the purposes of the study, participants who had used antibiotics to treat self-diagnosed disorders or symptoms or used leftover prescribed antibiotics without consulting a prescriber in the previous 12 months were considered to have practised SMA.

Data collectors were recruited and oriented to the digital data collection questionnaires using supplied Android smartphones. A detailed description of the Swahili translated consent form was given to all potential participants. The consenting participant in each household was recruited and interviewed for up to 15 min; antibiotics that are commonly available in the Dodoma region were shown to the respondents to guide them in remembering the antibiotics they had used or bought in the previous year.

### Data analysis

The collected data were downloaded from KoBo Collect software in Excel format, cleaned and then exported to R statistical software version 3.4.4 [15] for analysis. SMA was used as an outcome variable, and univariate and multivariate analyses were performed using various predictor variables. Univariate analysis involved the calculation of measures of central tendency, and multivariate analyses were performed to determine the independent effect of predictor variables on the probability of SMA across the rural and urban participants.

A descriptive analysis of the demographic information and various predictors is presented in tabular format, while the frequency of complaints leading to SMA is presented graphically. Finally, regression models to determine the odds of SMA with various predictors are presented for both crude odds ratios (univariate analyses) and adjusted odds ratios (multivariate analyses).

## Results

### Sociodemographic characteristics and prevalence of SMA among participants in Chemba District Council (rural) and Dodoma City Council (urban)

A total of 430 participants, one from each randomly selected household, were interviewed; of these, 62.6% (269/430) were from Dodoma City Council. The overall response rate was lower for participants in Chemba District Council (90.4%; 161/178) than for participants in Dodoma City Council (91.8%; 269/293). The majority (> 65%) of the participants in both the rural and urban localities were female. With regard to age distribution, the median age of the participants in the rural district was 36 (SD = 13.7), which was lower than the median age of 40 (SD = 12.8) among the participants in the urban district. The majority of the respondents in both the rural (76.4%; 123/161) and urban (82.2%; 221/269) districts had a primary school education; regarding marital status, 82.6% (133/161) and 87.4% (235/269) in the rural and urban localities, respectively, were single. The majority of the participants in the rural setting were farmers (74.5%; 120/161), which was slightly higher than the percentage in the urban setting, where 68.8% (185/269) reported that farming was their main economic activity. Additionally, approximately one-third of the participants in the urban district were small business owners (33.8%; 91/269), which was higher than the percentage in the rural district (13%; 21/161). The proportion of participants with health insurance was higher in rural district (22.4%; 36/161) than in the urban district (11.2%; 30/269).

Regarding the perceived distance from the household to a health care facility (hospital/health centre/dispensary) or a community drug outlet, the majority (76.4%; 123/161) of the participants in the rural setting perceived that they lived closer to a health care facility than a community drug outlet. Among the participants in the urban district, 57.2% (154/269) reported a shorter perceived distance from their household to a health care facility. The proportion of participants reporting awareness of the concept of antibiotic resistance and the associated complications was higher in the rural district (38.5%; 62/161) than in the urban district (26.4%; 71/269) (Table 1).

**Table 1:** Sociodemographic characteristics and prevalence of SMA among participants in the rural and urban settings (N = 430)

Variables	Rural (n = 161)		Urban (n = 269)	
	n (%)	Prevalence of SMA n (%): 38 (23.6)	N (%)	Prevalence of SMA n (%): 63 (23.4)
*Sex*				
Female	119 (73.9)	30 (78.9)	183 (68)	43 (68.3)
Male	42 (23.1)	8 (21.1)	86 (32)	20 (31.7)
*Age (years)*				
Mean	38.9	–	43.2	–
Median	36	–	40	–
sd	13.7	–	12.8	–
*Education*				
None	11 (6.8)	1 (2.6)	7 (2.6)	2 (3.2)
Primary school	123 (76.4)	30 (78.9)	221 (82.2)	43 (68.3)
Secondary +	27 (16.8)	7 (18.4)	41 (15.2)	18 (28.6)
*Marital status*				
Single	133 (82.6)	27 (71.1)	235 (87.4)	52 (82.5)
Married	18 (11.2)	8 (21.1)	22 (8.2)	10 (15.9)
Widowed	10 (6.2)	3 (7.8)	12 (4.5)	1 (1.6)
*Occupation*				
Farmer	120 (74.5)	29 (79.3)	185 (68.8)	28 (44.4)
Livestock and farming	20 (12.4)	1 (2.6)	24 (8.9)	7 (11.1)
Small business	21 (13)	5 (13.2)	91 (33.8)	32 (50.8)
Employed	21 (13)	6 (15.8)	16 (5.9)	7 (11.1)
*Subscription to health insurance*				
Yes	36 (22.4)	7 (18.4)	30 (11.2)	7 (11.1)
No	125 (77.6)	31 (81.6)	239 (88.8)	56 (88.9)
*Perceived proximity to drug outlet and health facility*				
Drug outlet	38 (23.6)	34 (89.5)	115 (42.8)	43 (68.3)
Health centre	123 (76.4)	4 (10.5)	154 (57.2)	20 (31.7)
*Awareness on antibiotic resistance*				
Yes	62 (38.5)	14 (36.8)	71 (26.4)	17 (27)
No	99 (61.5)	24 (63.2)	198 (73.6)	46 (73)
*Frequency of SMA*				
None	1 (0.62%)	-	1 (0.37%)	-
1–2	147 (91.3%)	-	238 (88.48%)	-
3–4	13 (8.07)	-	30 (11.15%)	-

Generally, 23.5% (101/430) of the participants in both the rural and urban districts reported having practised SMA within the previous year, with 23.6% (38/161) in the rural district and 23.4% (63/269) in the urban district. The median frequency of SMA among both the rural and urban participants was 2 times.

### Complaints leading to SMA and antibiotics frequently used

**Fig. 1:**
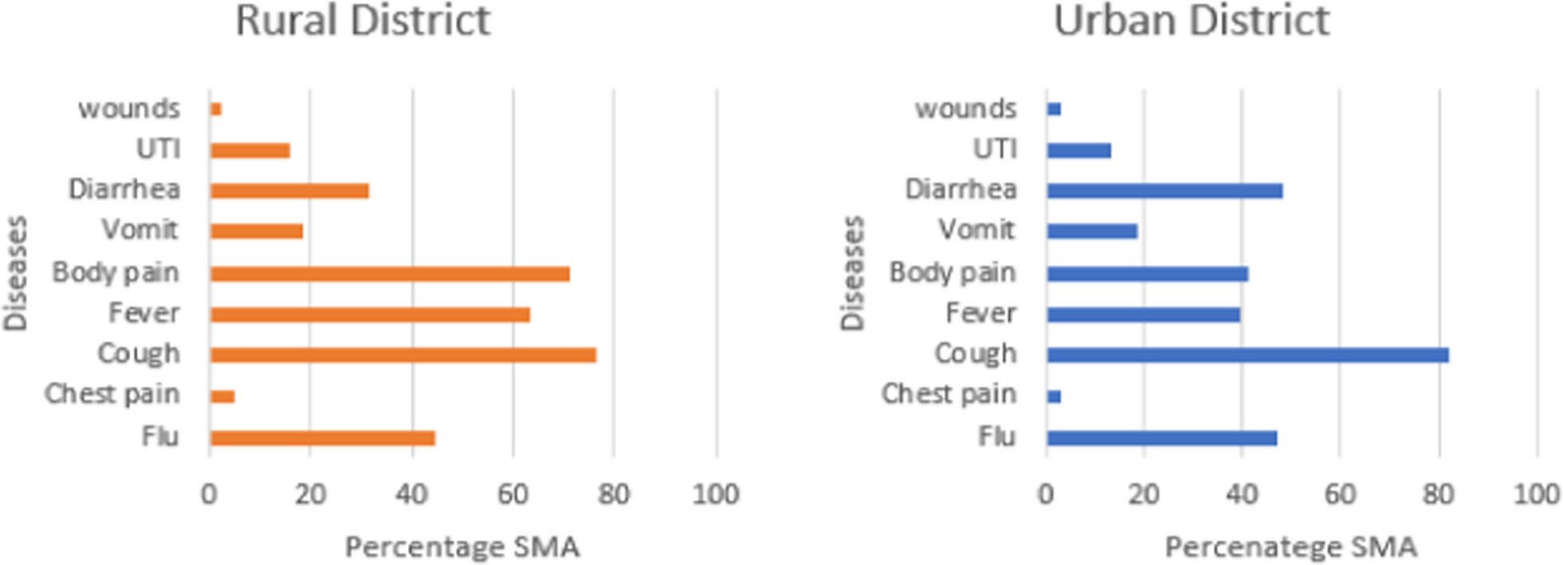
Complaints prompting SMA in the rural district (left) and urban district (right)

Cough complaints accounted for the majority of SMA practices among both the rural and urban participants (76.3% and 82%, respectively). Other commonly reported complaints prompting SMA were, ordered by proportion, body pain (71.1%), fever (63.2%), flu (44.7%) and diarrhoea (31.3%) in the rural district, whereas diarrhoea (48.2%), flu (47.1%), body pain (41.5%) and fever (39.7%) were the complaints that prompted SMA in the urban district (Fig. 1/Appendix 1).

**Fig. 2:**
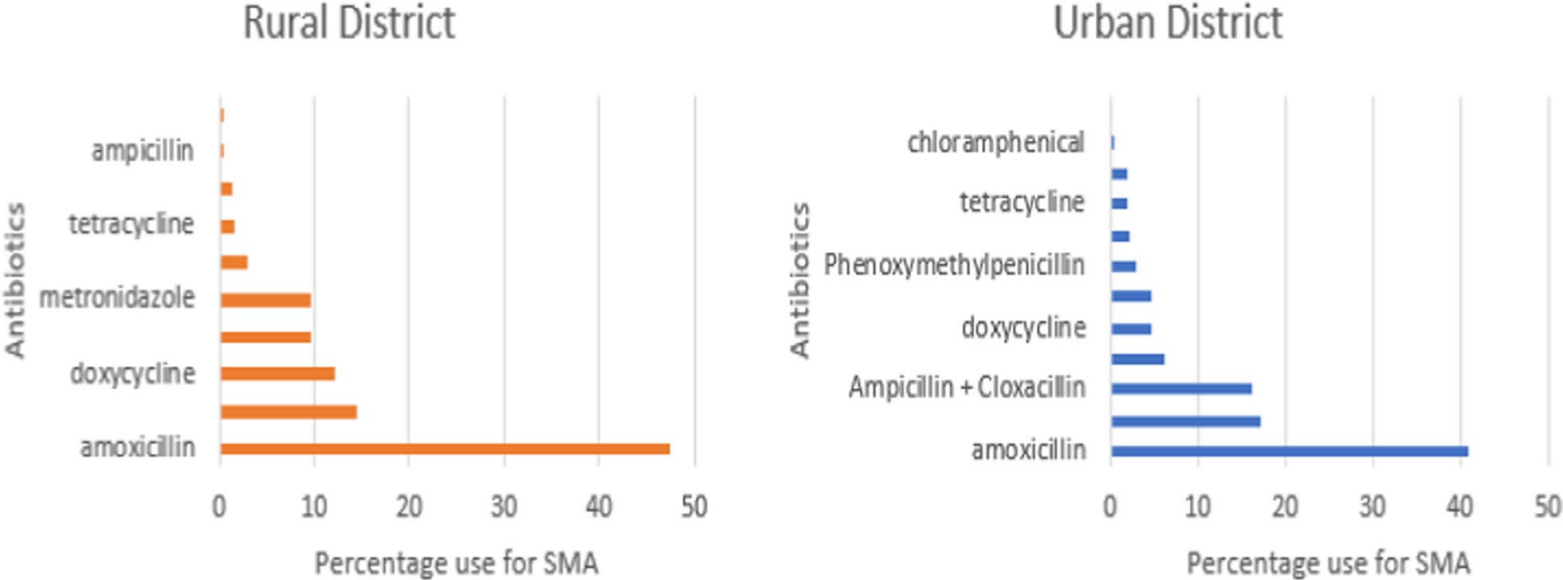
Antibiotics for SMA in the rural district (left) and urban district (right)

Generally, a larger number of antibiotic varieties was used by the urban participants (13 antibiotics) than by their rural counterparts (9 antibiotics) (Fig. 2/Appendix 2). The most commonly used antibiotic for self-medication among both the rural (47.3%) and urban (40.9%) participants was amoxicillin. Other antibiotics commonly consumed through self-medication in the two settings (rural vs. urban) were ampicillin + cloxacillin fixed drug combination (14.6% vs. 16.2%), doxycycline (12.1% vs. 4.8%) metronidazole (9.6% vs. 17.2%) and cotrimoxazole (9.6% vs. 6.1%) (Fig. 2/Appendix 2).

### Predictors of SMA in the rural and urban settings

#### Univariate logistic regression model

In the univariate model (Table 2), there was a comparable increase in the odds of SMA among participants with single marital status in both settings; compared to those in the married category, single participants were 1.27 times (crude OR: 1.273; 95% CI: 1.030, 1.567; *p* = 0.0237) and 1.26 times (crude OR: 1.262; 95% CI: 1.051, 1.517; *p* = 0.013) more likely to practise SMA in the rural and urban districts, respectively. Compared to the nonfarming participants in this study, the odds of SMA decreased by 23.3% (crude OR: 0.767; 95% CI: 0.691, 0.852; *p* < 0.001) among farmers in the urban setting, while the likelihood of SMA among farmers in the rural setting was 0.022 times (crude OR: 1.022; 95% CI: 0.879, 1.190; *p* = 0.775) higher; however, the latter observation was not statistically significant. Although the results were not statistically significant, the odds of SMA were 5.2% (crude OR: 0.948; 95% CI: 0.809, 1.110; *p* = 0.508) and 0.1% (crude OR: 0.999; 95% CI: 0.850, 1.174; *p* = 0.991) lower for participants with any form of health insurance than for participants lacking health insurance in the rural and urban settings, respectively.

**Table 2:** Predictors of self-medication with antibiotics in the rural and urban settings

Variables	Rural						Urban					
	Univariate model			Multivariate model			Univariate model			Multivariate model		
	cOR	95% CI	*p* value	aOR	95% CI	*p* value	cOR	95% CI	*p* value	aOR	95% CI	*p* value
*Sex*												
Female	Ref			Ref			Ref			Ref		
Male	0.94	0.81, 1.09	0.422	1.027	1.913, 3.33	0.556	0.998	0.895, 1.112	0.965	0.960	0.856, 1.077	0.492
Age	0.997	0.992, 1.00	0.2	0.999	0.995, 1.003	0.697	0.994	0.990, 0.998	0.198	0.997	0.992, 1.002	0.222
*Education*												
None	Ref			Ref			Ref			Ref		
Primary	1.165	0.896, 1.516	0.256	1.057	0.904, 1.236	0.485	0.912	0.667, 1.248	0.569	0.830	0.611, 1.126	0.231
Secondary or higher	1.183	0.877, 1.596	0.272	1.0105	0.831	0.916	1.166	0.835, 1.628	0.369	0.918	0.648, 1.300	0.630
*Marital status*												
Married	Ref			Ref			Ref			Ref		
Single	1.273	1.036, 1.567	0.0239	1.041	0.893, 1.21	0.605	1.262	1.051, 1.517	0.013	0.975	0.797, 1.192	0.805
Widowed	1.102	0.840, 1.445	0.4838	1.129	0.963, 1.324	0.136	0.871	0.682, 1.111	0.267	0.957	0.748, 1.222	0.723
*Occupation*												
Farming												
No	Ref			Ref			Ref			Ref		
Yes	1.022	0.879, 1.190	0.775	0.916	0.813, 1.030	0.146	0.767	0.691, 0.852	< 0.001	0.827	0.716, 0.955	0.010
*Farming and livestock*												
No	Ref						Ref			Ref		
Yes	0.809	0.664, 0.985	0.0365	0.957	0.821, 1.114	0.573	1.065	0.891, 1.272	0.488	1.017	0.818, 1.264	0.878
*Business*												
No	Ref						Ref			Ref		
Yes	1.002	0.824, 1.219	0.981	0.932	0.827, 1.051	0.253	1.194	0.074, 1.327	0.0001	1.096	0.975, 1.232	0.126
*Employed*												
No	Ref						Ref			Ref		
Yes	1.059	0.871, 1.288	0.568	1.06	0.936, 1.2001	0.361	1.241	1.002, 1.536	0.048	1.133	0.873, 1.471	0.348
*Subscription to health insurance*												
No	Ref			Ref			Ref			Ref		
Yes	0.948	0.809, 1.110	0.508	0.935	0.848, 1.032	0.184	0.999	0.850, 1.174	0.991	0.925	0.766, 1.116	0.416
*Perceived distance to a health care centre or drug outlet*												
Drug outlet	Ref			Ref			Ref			Ref		
Health facility	0.422	0.390, 0.457	< 0.001	0.421	0.388, 0.458	< 0.001	0.783	0.710, 0.864	< 0.001	0.837	0.755, 0.929	< 0.001
*Awareness of antibiotic resistance*												
No	Ref			Ref			Ref			Ref		
Yes	0.984	0.859, 1.126	0.811	1.06	0.984, 1.141	0.123	1.007	0.897, 1.130	0.904	0.936	0.832, 1.053	0.270

*cOR*  crude odds ratio

*aOR*  adjusted odds ratio

With regard to the perceived distance to a community drug outlet or a health care facility, the participants perceiving a shorter distance to a health care facility had a 57.8% lower likelihood of SMA in the rural setting (crude OR: 0.422; 95% CI: 0.390, 0.457; *p* < 0.001) and a 21.7% lower likelihood in the urban setting (crude OR: 0.783; 95% CI: 0.710, 0.864; *p* < 0.001) compared to those perceiving a shorter distance to a community drug outlet.

### Multivariate logistic regression model

The participants who reported living closer to a health care facility than a drug outlet had 57.9% (adjusted OR: 0.421; 95% CI: 0.388, 0.458; *p* < 0.001) and 16.3% (adjusted OR: 0.837; 95% CI: 0.755, 0.929; *p* < 0.001) lower odds of SMA than those who reported living closer to a drug outlet in the rural and urban districts, respectively. Compared to other occupations in this study, the odds of SMA among the farming participants decreased by 17.3% (adjusted OR: 0.827; 95% CI: 0.716, 0.955; *p* = 0.01) and 8.4% (adjusted OR: 0.916; 95% CI: 0.813.1.030; *p* = 0.146) in the urban and rural districts, respectively (Table 2).

## Discussion

The main objective of this study was to establish the prevalence and predictors of SMA in rural and urban communities of the Dodoma region, Tanzania. We report a comparable prevalence of SMA across the rural and urban districts and decreased odds of SMA among farmers compared to participants in other occupations, those with health insurance compared to the uninsured, and those living closer to a health care facility compared to those living closer to a drug outlet.

The overall prevalence of SMA among the rural and urban respondents in this study was similar. This observation is incongruent with the results reported by studies in India, where higher SMA was found among urban respondents than among their rural counterparts [9]. In another study, more SMA was found among respondents in rural than in urban localities [18]. Our study, therefore, reveals the need to focus equally on strategies to reduce SMA practices in both rural and urban settings.

Furthermore, the most commonly used antibiotic for self-medication in the two districts (rural vs. urban) was amoxicillin (47.3% vs. 40.9%), and others were ampicillin + cloxacillin fixed drug combination (14.6% vs. 16.2%), metronidazole (9.6% vs. 17.2%), doxycycline (12.1% vs. 4.8%) and cotrimoxazole (9.6% vs. 6.1%). This finding is congruent with the findings of studies in Asmara Eritrea, Northern Tanzania, Northeastern Ethiopia and Peru, where amoxicillin was one of the most common antibiotics used for self-medication [3, 9, 19, 20]. Amoxicillin, metronidazole, doxycycline and cotrimoxazole are classified in the “*Access, Watch and Reserve—AWaRe*” access group in the Tanzanian Standard Treatment Guideline (STG) [21] and thus are stocked at almost all health care levels, including community drug outlets, hence increasing the risk of their misuse and overuse.

Participants from both the rural and urban localities who reported a longer perceived distance from their household to a community drug outlet and a shorter perceived distance to a health care facility (dispensary/health centre/hospital) were less likely to practise SMA. This finding is comparable to another study in Northern Tanzania, where high SMA practices were reported among people living near community drug outlets [3]. In other words, participants living closer to a community drug outlet were more at risk of practising SMA, partly because antibiotics for self-medication can easily be accessed and purchased from these poorly regulated community drug outlets [3]. Initiatives to contain irresponsible dispensing across community drugstores as well as antibiotic stewardship campaigns involving health care providers, community drug dispensers and the community at large are therefore imperative.

The majority of the respondents from both the rural and urban settings in this study were farmers, who had the lowest odds of SMA compared to participants in other occupations (employed, business, and livestock keeping and farming). This finding may have resulted from the fact that the majority of the farming participants resided in low-population-density areas where households are scattered far from each other with a lower number of community drug outlets and the presence of at least a nearby dispensary or health centre, reinforcing the observation that a longer distance from a household to a drug outlet reduces the risk of SMA [3]. This information is especially valuable to policy makers because it reveals the need to institute more robust laws and regulations on transactions in antibiotics in community drug outlets.

Although the results were not statistically significant, participants with health insurance in both the rural and urban settings were less likely to practise SMA than those who were uninsured; this observation is similar to the findings of a study in Kenya [22]. It is equally important for campaigns to be held to encourage people to subscribe to health insurance schemes, especially in developing countries such as Tanzania, where infectious diseases are prevalent and the majority of people cannot afford to pay cash for medical services [23]. Nevertheless, with an increase in the population with health insurance, the need for prescribers to adhere to standard treatment guidelines and avoid overprescribing antibiotics is inevitable.

Our study provides insight into SMA practices in both rural and urban settings for appropriate interventions in the fight against antimicrobial resistance (AMR) in two socially and economically different communities.

The limitation of this study is that data collection was performed during the day (morning to early evening), leaving the possibility of missing household members who might have left early in the morning. Additionally, for respondents who did not remember the antibiotics they had used, we showed them a variety of antibiotics for identification; this is a challenge because some medications might have a similar appearance yet belong to different therapeutic groups.

## Conclusions

In our study, the prevalence of SMA was almost comparable in the rural and urban districts; in both cases, a low risk of SMA was seen among farmers compared to nonfarming participants. A higher proportion of participants in the rural district reported a shorter perceived distance to a health care facility than a community drug outlet than those in the urban district. In this study, a shorter perceived distance from a household to a community drug outlet than to a health care facility increased the risk of SMA. There was a low prevalence of SMA among participants with health insurance in both the rural and urban districts. Tailored community antibiotic stewardship campaigns on the dangers of antibiotic resistance as well as enforcing existing laws and enacting others to regulate the conduct of drug dispensers regarding the sale of antibiotics in community drug outlets are needed.

This study therefore provides information about the need to focus equally on strategies to reduce SMA practices in both rural and urban settings and adds to the specific set of predictors to focus on in such an effort, thus opening more directions from which to address antimicrobial resistance.

### Supplementary Information

**Additional file 1: Figure 1**. Complaints prompting SMA in the rural district (left) and urban district (right).

**Additional file 2: Figure 2**. Antibiotics for SMA in the rural district (left) and urban district (right).

## Data Availability

The data used in this manuscript are available upon reasonable request from the corresponding author.

## Appendix 1

See Fig. 1

## Appendix 2

See Fig. 2

## Abbreviations

NM-AIST: Nelson Mandela African Institution of Science and Technology
UDOM: The University of Dodoma
WHO: World Health Organization
SMA: Self-medication with antibiotics
AMR: Antimicrobial resistance
ODK: Open Data Kit
