# Supplementary material for: Prevalence and predictors of self-medication with antibiotics in selected urban and rural districts of the Dodoma region, Central Tanzania: a cross-sectional study
Source: Antimicrob Resist Infect Control. 2022 Jun 16;11:86. doi: 10.1186/s13756-022-01124-9 (PMC9205028; doi:10.1186/s13756-022-01124-9)
Supplement: Supplementary file 1 — Additional file 1: Figure 1. Complaints prompting SMA in the rural district (left) and urban district (right). [file 13756_2022_1124_MOESM1_ESM.docx]

QUESTIONNAIRE: ENGLISH VERSION

| **PART A: GENERAL INFORMATION** | | |
| --- | --- | --- |
| ***District*** | ***Ward*** | ***Village /Street*** |
| ***Household number*** | | |
| ***Date of Visit*** | | |
| ***Name of data collector*** | | |

| **PART B: DEMOGRAPHIC INFORMATION** | | | |
| --- | --- | --- | --- |
| **S/N** | **Questions/Item** | **Response** | **Tick** |
| 1. | Age of respondent (Years) |  | |
| 2. | Sex | 1. Male |  |
|  |  | 1. Female |  |
| 3. | Marital status | 1. Single |  |
|  |  | 1. Married |  |
|  |  | 1. Divorced |  |
|  |  | 1. Widowed |  |
| 4. | What is the level of your education? | 1. No formal education |  |
|  |  | 1. Primary school education |  |
|  |  | 1. Secondary School education |  |
|  |  | 1. College education |  |
|  |  | 1. Others (specify) |  |
| 5. | What is your occupation? | 1. Farming |  |
|  |  | 1. Livestock keeping |  |
|  |  | 1. Farming and livestock keeping |  |
|  |  | 1. Employed |  |
|  |  | 1. Others (*specify*) |  |
| 6. | Between a health care facility and a community drug outlet, which one is closer to where you stay? | 1. Health care facility |  |
|  |  | 1. Community drug outlet |  |

| **PART C: SELF MEDICATION BEHAVIORS** | | | |
| --- | --- | --- | --- |
| 1. | Have you ever taken antibiotics? | A. Yes |  |
|  |  | B. No |  |
| 2. | Have you ever treated yourself (self-medicated) with antibiotics? | A. Yes |  |
|  |  | B. No |  |
| 3. | How many times did you treat yourself with antibiotics in the past one year? |  |  |
| 4. | What was (were) your reason(s) of self-medication with antibiotics? (check more than one if applicable)   \|  \| \| --- \| | A. Cost saving |  |
|  |  | B. Convenience |  |
|  |  | C. Lack of trust in prescribing doctor |  |
|  |  | D. Others (specify) |  |
|  | In which seasons of the year do you use antibiotics often? | 1. Rainy seasons |  |
|  |  | 1. Dry seasons |  |
| 5. | For which of the following complaint(s) did you use antibiotics? (check more than one if applicable) | A. Runny nose |  |
|  |  | B. Nasal congestion |  |
|  |  | C. Cough |  |
|  |  | D. Sore throat |  |
|  |  | E. Fever |  |
|  |  | F. Aches and pains |  |
|  |  | G. Vomiting |  |
|  |  | H. Diarrhea |  |
|  |  | I. Skin wounds |  |
|  |  | J. Others (specify) |  |
|  |  |  |  |
| 6. | Your selection of antibiotics was based on… (check more than one if applicable) | A. Recommendation by community pharmacists |  |
|  |  | B. Opinion of family members |  |
|  |  | C. Opinion of friends |  |
|  |  | D. My own experience |  |
|  |  | F. Previous doctor’s prescription |  |
| 7. | What did you consider when selecting antibiotics? (check more than one if applicable) | A. Type of antibiotics |  |
|  |  | B. Brand of antibiotics |  |
|  |  | C. Price of antibiotics |  |
|  |  | D. Indications for use |  |
|  |  | E. Adverse reactions |  |
|  |  | F. Others (specify) |  |
| 8. | Where did you usually obtain antibiotics from for self-medication? (check more than one if applicable) | A. Community pharmacies |  |
|  |  |  |  |
|  |  | C. Leftover from previous prescription |  |
|  |  | D. Friends and relatives |  |
|  |  | E. Others (specify) |  |
| 16. |  |  |  |
| 17. | When did you normally stop taking antibiotics? (check more than one if applicable) | A. After a few days regardless of the outcome |  |
|  |  | B. After symptoms disappeared |  |
|  |  | C. A few days after the recovery |  |
|  |  | D. After antibiotics ran out |  |
|  |  | E. At the completion of the course |  |
|  |  | F. After consulting a doctor/pharmacist |  |
|  |  | G. Others (specify) |  |
| 23. | Are you aware of antibiotic resistance and its dangers? | A. Yes |  |
|  |  | B. No |  |
| 24. | Please write down the names of antibiotics you have ever taken for SELF-MEDICATION: | 1. | |
|  |  | 2. | |
|  |  | 3. | |
|  |  | 4. | |
|  | How many different antibiotics did you take maximally during a single illness? |  | |
