# Supplementary material for: Prevalence and predictors of self-medication with antibiotics in selected urban and rural districts of the Dodoma region, Central Tanzania: a cross-sectional study
Source: Antimicrob Resist Infect Control. 2022 Jun 16;11:86. doi: 10.1186/s13756-022-01124-9 (PMC9205028; doi:10.1186/s13756-022-01124-9)
Supplement: Supplementary file 2 — Additional file 2: Figure 2. Antibiotics for SMA in the rural district (left) and urban district (right). [file 13756_2022_1124_MOESM2_ESM.docx]

KISWAHILI QUESTIONNAIRE: DODOSO NAMBARI MOJA

| **SEHEMU A: MAELEZO YA JUMLA/AWALI** | | | | | | | |
| --- | --- | --- | --- | --- | --- | --- | --- |
| ***Wilaya:*** | | ***Kata:*** | | ***Kijiji /Mtaa:*** | | | |
| ***Numbari ya Nyumba:*** | |  | | | | | |
| ***Tarehe ya Kutembelewa:*** | |  | | | | | |
| ***Jina la Mchukuua taarifa:*** | |  | | | | | |
| **SEHEMU B: TAKWIMU ZA WAKAZI** | | | | | | | |
| **Na** | **Maswali** | | **Jibu** | | | **Alama ya vema** | |
| 1. | UmriwaMhojiwa (miaka) | | 1. 18 - 34 | | |  | |
|  |  |  | 1. 35 - 49 | | |  | |
|  |  |  | 1. 50 – 70 | | |  | |
|  |  |  | 1. >70 | | |  | |
| 2. | Jinsia | | 1. Mwanaume | | |  | |
|  |  |  | 1. Mwanamke | | |  | |
| 3. | Hali yandoa | | 1. Sijaoa/olewa | | |  | |
|  |  |  | 1. Nimeoa/olewa | | |  | |
|  |  |  | 1. Mtalikiwa | | |  | |
|  |  |  | 1. Mgane/Mjane | | |  | |
| 4. | Una kiwango gani cha elimu? | | 1. Sina elimu rasmi | | |  | |
|  |  |  | 1. Elimu ya msingi | | |  | |
|  |  |  | 1. Elimu ya sekondari | | |  | |
|  |  |  | 1. Elimu Elimu ya chuo | | |  | |
|  |  |  | 1. Nyingine (itaje) | | |  | |
| 5. | Unafanya kazi gani? | | 1. Ukulima/Mkulima | | |  | |
|  |  |  | 1. Ufugaji | | |  | |
|  |  |  | 1. Mkulima na Mfugaji | | |  | |
|  |  |  | 1. Nimeajiriwa | | |  | |
|  |  |  | 1. Nyingine (*taja*) | | |  | |
| 6. | Kati ya kituo cha huduma ya afya na duka la dawa,k ipi kipo karibu na unapoishi? | | 1. Kituo cha huduma ya afya | | |  | |
|  |  |  | 1. Duka la dawa | | |  | |
| **SEHEMU C: TABIA YA KUJIAMULIA KUTUMIA DAWA** | | | | | | | |
| 1. | Umewahi kutumia dawa ya kuua vijisumu? | | A.Ndio : | | B.Hapana: | | |
| 2. | Je! Umewahi kujitibu na dawa yakuua vijisumu? | | A.Ndio | | B. Hapana | | |
| 3. | Mara ngapi umejitibu na dawa ya kuua vijisumu katika mwaka mmoja uliopita? | |  | |  | | |
| 4. | Nini ilikuwa (zilikuwa) sababu zako zakujitibu na dawa ya kuua vijisumu? (weka jibu zaidi ya moja kama inafaa) | | A. Kupunguza gharama | |  | | |
|  |  | | B. Urahisi | |  | | |
|  |  | | C. Kukosa imani na daktari | |  | | |
|  |  | | D. Nyingine (taja) | |  | | |
| 5. | Je, kwalalamiko gani ulitumia dawa ya kuua vijisumu? (weka jibu Zaidi ya moja kama inafaa) | | A. Mafua ya kutiririka | |  | | |
|  |  |  | B. Pua kubanwa na mafua | |  | | |
|  |  |  | C. Kikohozi | |  | | |
|  |  |  | D. Maumivu ya koo | |  | | |
|  |  |  | E. Homa | |  | | |
|  |  |  | F. Maumivu | |  | | |
|  |  |  | G. Kutapika | |  | | |
|  |  |  | H. Kuhara | |  | | |
|  |  |  | I. Majeraha ya ngozi | |  | | |
|  |  |  | J.Nyingine (taja) | |  | | |
| 6. | Uchaguzi wako wa dawa yakuua vijisumu ulizingatia ... (wekajibu Zaidi yamojakamainafaa) | | A. Mapendekezo kutoka kwa mfamasia wa jamii | |  | | |
|  |  |  | B. Maoni ya wanafamilia | |  | | |
|  |  |  | C. Maoni ya marafiki | |  | | |
|  |  |  | D. Uzoefu binafsi | |  | | |
|  |  |  | E. Mapendekezo ya wananchi | |  | | |
|  |  |  | F. Dawa kutoka kwa daktari wa awali | |  | | |
| 7. | Ulizingatia nini wakati wakuchagua dawa yakuua vijisumu? (weka jibu zaidi ya moja kama inafaa) | | A. Aina ya dawa ya kuua vijisumu | |  | | |
|  |  |  | B. Chapa ya dawa yakuua vijisumu | |  | | |
|  |  |  | C. Gharama ya dawa ya kuua vijisumu | |  | | |
|  |  |  | D. Vielelezo vya matumizi | |  | | |
|  |  |  | E. Athari mbaya | |  | | |
|  |  |  | F.Nyingine (taja) | |  | | |
| 8. | Huwa unapata wapi dawa ya kuua vijisumu kwaajili ya kujitibu (weka jibu zaidi ya moja kama inafaa) | | A. Famasi ya jamii | |  | | |
|  |  |  | B. Watendaji wa TCM | |  | | |
|  |  |  | C. Mabaki ya dawa zilizopita | |  | | |
|  |  |  | D. manunuzi kwa mtandao/E-maduka ya dawa | |  | | |
|  |  |  | E. Nyingine(taja) | |  | | |
| 9. | Wakati gani unasitisha kutumia dawa ya kuua vijisumu? (wekajibu Zaidi yamoja kama inafaa) | | A.Baada ya siku chache bila kujali matokeo | |  | | |
|  |  | | B. Baada ya dalili kutoweka | |  | | |
|  |  | | C. Siku chache baada ya kupona | |  | | |
|  |  | | D. Baada ya dawa ya kuua vijisumu kuisha | |  | | |
|  |  | | E.Wakati wakukamilika kwa kozi | |  | | |
|  |  | | F. Baada ya kushaurianana daktari / mfamasia | |  | | |
| 10 | Umewahi kusikia usugu wa vijisumu? | | A. Ndio | | B. Hapana | | |
| 11 | Tafadhali orodhesha majina ya dawa yakuua vijisumu uliyowahi kutumia kwa KUJITIBU: | | 1. | |  | | |
|  |  | | 2. | |  | | |
|  |  | | 3. | |  | | |
|  |  | | 4. | |  | | |
| **SEHEMU D: KAYA NA HALI YA UCHUMI** | | | | | | | |
| 1. | 1. Wana kaya wangapi wana umri wa miaka 18 au chini ya hapo? | | A. Sita au zaidi | | | |  |
|  |  |  | B. Watano | | | |  |
|  |  |  | C. Wanne | | | |  |
|  |  |  | D. Watatu | | | |  |
|  |  |  | E. Wawili | | | |  |
|  |  |  | F. Mmoja | | | |  |
|  |  |  | G. Hakuna | | | |  |
| 2. | Wanakaya wangapi wana miaka kati ya 6 na 18 wanasoma kwasasa? | | A. Hapana | | | |  |
|  |  |  | B. Ndio | | | |  |
|  |  |  | C. Hakuna mwanakaya waumri kati ya 6 na 18 | | | |  |
| 3 | Vifaa gani vikuu vya ujenzi vilivyo tumika kwenye jengo kuu? | | AMatofali ya kuchoma | | | |  |
|  |  |  | B. Miti na udongo, nyasi, matofali ya kukaushwa na jua au nyingine | | | |  |
|  |  |  | C. Mawe, Matofali ya saruji au mbao | | | |  |
| 4. | Vifaa gani vikuu vya ujenzi vinazotumika kwa paa ya jingo kuu? | | A. Nyasi / majani, udongo na majani, au nyingine | | | |  |
|  |  |  | B. Mabati,matofali, saruji, au asbestosi | | | |  |
| 5 | Ni nishati gani kuu hutumiwa kwa kupika? | | 1. Kuni, makaa ya mawe, nishati ya jua, nishati ya gesi, mabaki ya kuni/shamba, au mabaki ya wanyama | | | |  |
|  |  |  | B. Mkaa, mafuta, gesi (viwanda), umeme, jenereta / chanzo binafsi, au nyingine | | | |  |
| 6. | Je! Nyumba yako ina luninga? | | A. Hapana | | | |  |
|  |  |  | B. Ndio | | | |  |
| 7. | Je! Nyumba yako ina radio yoyote, kanda / rekodi za tepe, au mifumo ya hi-fi? | | A. Hapana | | | |  |
|  |  |  | B. Ndio | | | |  |
| 8. | Je, nyumba yako ina taa yoyote? | | A. Hapana | | | |  |
|  |  |  | B. Ndio | | | |  |
| 9. | Je, nyumba yako ina meza yoyote? | | A. Hapana | | | |  |
|  |  |  | B. Ndio | | | |  |
| 10. | Ikiwa kaya ililima mazao yoyote katika miezi 12 iliyopita, je, kwasasa inamiliki ng'ombe dume,ngombe jike,ndama jike, ndama dume,ng'ombe maksai, ng'ombe? | | A. Hakuna mazao, wala hakuna ng'ombe | | | |  |
|  |  |  | B. Hakuna mazao, na ng'ombe | | | |  |
|  |  |  | C. Kuna mazao, lakini hakuna ng'ombe | | | |  |
|  |  |  | D. Kuna mazao na ng’ombe | | | |  |
